# Functionally Relevant Maculopathy and Optic Atrophy in Spinocerebellar Ataxia Type 1

**DOI:** 10.1002/mdc3.12949

**Published:** 2020-05-06

**Authors:** Frederike Cosima Oertel, Oliver Zeitz, Maria Rönnefarth, Charlotte Bereuter, Seyedamirhosein Motamedi, Hanna G. Zimmermann, Joseph Kuchling, Anne Sophie Grosch, Sarah Doss, Andrew Browne, Friedemann Paul, Tanja Schmitz‐Hübsch, Alexander U. Brandt

**Affiliations:** ^1^ Experimental and Clinical Research Center, Max‐Delbrück‐Centrum für Molekulare Medizin and Charité–Universitätsmedizin Berlin, corporate member of Freie Universität Berlin Humboldt‐Universität zu Berlin, and Berlin Institute of Health Berlin Germany; ^2^ NeuroCure Clinical Research Center, Charité–Universitätsmedizin Berlin, corporate member of Freie Universität Berlin Humboldt‐Universität zu Berlin, and Berlin Institute of Health Berlin Germany; ^3^ Department of Ophthalmology, Charité–Universitätsmedizin Berlin, corporate member of Freie Universität Berlin Humboldt‐Universität zu Berlin, and Berlin Institute of Health Berlin Germany; ^4^ Department of Neurology, Charité–Universitätsmedizin Berlin, corporate member of Freie Universität Berlin Humboldt‐Universität zu Berlin, and Berlin Institute of Health Berlin Germany; ^5^ Department of Neurological Sciences University of Nebraska Medical Center Nebraska Omaha USA; ^6^ Department of Ophthalmology University of California Irvine Irvine California USA; ^7^ Department of Neurology University of California Irvine Irvine California USA

**Keywords:** SCA‐ATXN1, SCA1, optical coherence tomography, optic atrophy, maculopathy

## Abstract

**Background:**

Spinocerebellar ataxia type 1 (SCA‐ATXN1) is an inherited progressive ataxia disorder characterized by an adult‐onset cerebellar syndrome combined with nonataxia signs. Retinal or optic nerve affection are not systematically described.

**Objectives:**

To describe a retinal phenotype and its functional relevance in SCA‐ATXN1.

**Methods:**

We applied optical coherence tomography (OCT) in 20 index cases with SCA‐ATXN1 and 22 healthy controls (HCs), investigating qualitative changes and quantifying the peripapillary retinal nerve fiber layer (pRNFL) thickness and combined ganglion cell and inner plexiform layer (GCIP) volume as markers of optic atrophy and outer retinal layers as markers of maculopathy. Visual function was assessed by high‐ (HC‐VA) and low‐contrast visual acuity (LC‐VA) and the Hardy‐Rand‐Rittler pseudoisochromatic test for color vision.

**Results:**

Five patients (25%) showed distinct maculopathies in the ellipsoid zone (EZ). Furthermore, pRNFL (*P* < 0.001) and GCIP (*P* = 0.002) were reduced in patients (pRNFL, 80.86 ± 9.49 μm; GCIP, 1.84 ± 0.16 mm^3^) compared with HCs (pRNFL, 97.02 ± 8.34 μm; GCIP, 1.98 ± 0.12 mm^3^). Outer macular layers were similar between groups, but reduced in patients with maculopathies. HC‐VA (*P* = 0.002) and LC‐VA (*P* < 0.001) were reduced in patients (HC‐VA [logMAR]: 0.01 ± 010; LC‐VA [logMAR]: 0.44 ± 0.16) compared with HCs (HC‐VA [logMAR]: –0.12 ± 0.08; LC‐VA [logMAR]: 0.25 ± 0.05). Color vision was abnormal in 2 patients with maculopathies.

**Conclusions:**

A distinct maculopathy, termed EZ disruption, as well as optic atrophy add to the known nonataxia features in SCA‐ATXN1. Whereas optic atrophy may be understood as part of a widespread neurodegeneration, EZ disruption may be explained by effects of ataxin‐1 gene or protein on photoreceptors. Our findings extend the spectrum of nonataxia signs in SCA‐ATXN1 with potential relevance for diagnosis and monitoring.

Spinocerebellar ataxia type one (SCA‐ATXN1 in revised nomenclature, formerly SCA1) is a dominantly inherited autosomal progressive ataxia disorder caused by translated CAG‐repeat expansions in the ataxin 1 gene (ATXN1).^1^, ^2^, ^3^ The elongated ATXN1 protein seems to be not only neurotoxic itself, but several conserved sequence motifs in the ATXN1 protein indicate a role of ATXN1 as a regulator of transcription and RNA processing, suggesting widespread pathological consequences in SCA‐ATXN1.^4^ Clinically, SCA‐ATXN1 is typically characterized by an adult‐onset cerebellar syndrome, which is typically combined with nonataxia signs.^5^, ^6^ These include most prominently pyramidal signs, but also comprise brainstem oculomotor signs, autonomic dysfunction, late cognitive impairment, and a variety of movement disorders, such as chorea, dystonia, myoclonus, and tremor.^7^, ^8^, ^9^, ^10^ Retinal or optic nerve affection are well‐established clinical characteristics of several spinocerebellar ataxias, such as spinocerebellar ataxia type 7 (SCA‐ATXN7), but not yet described in SCA‐ATXN1.^11^, ^12^ However, a few incidental case series and smaller studies suggest retinal neuroaxonal degeneration or report a potentially distinct maculopathy, including photoreceptor alterations, and a decline of visual function in a subset of SCA‐ATXN1 patients.^13^, ^14^ This is further sustained by drosophila animal models of SCA‐ATXN1 suggesting retinal neurodegeneration.^15^

Against this background, we aimed to investigate neurodegeneration of retina and optic nerve, macular anomalies, and their functional relevance in a cross‐sectional cohort of 20 index cases with SCA‐ATXN1.

## Methods

### Study Sample

Twenty patients with genetically confirmed and manifest SCA1‐ATXN1 were prospectively recruited between June 2010 and November 2016 for this cross‐sectional observational study from one university ataxia clinic (Charité–Universitätsmedizin Berlin, Berlin, Germany) and examined at the NeuroCure Clinical Research Center (NCRC). The inclusion criterion was genetically confirmed diagnosis of SCA1‐ATXN1. Exclusion criteria for this analysis included missing retinal optical coherence tomography (OCT) data. Two patients and three eyes in 2 other patients had OCT of insufficient image quality and were excluded from the statistical (but not from the descriptive) analysis. Nine patients (45%) were previously published in a smaller cohort study by Stricker et al., which reported OCT findings from an earlier time point.^14^ Number of CAG‐repeats, Scale for the Assessment and Rating of Ataxia (SARA) score^16^ within 90 days of the OCT examination and time since onset of permanent gait ataxia were accessible in the clinical database for 16, 15, and 20 patients, respectively (Table 1). Apart from the ataxia rating, a description of eye movements was available for 20 patients, from which 19 (95%) presented with abnormalities: 16 patients (80%) with saccadic pursuit, 10 patients (50%) with slowing of saccades, 11 patients (55%) with failure of fixation suppression in testing of the vestibular‐ocular reflex, 7 patients (35%) with hypo‐ or hypermetric saccades, and 3 patients (15%) with a gazed‐evoked nystagmus.

For comparison, we included cross‐sectional data of 22 healthy controls (HCs) from the NCRC's normative OCT database matched for sex (χ^2^ < 0.001; *P* > 0.999) and age (W = 200; *P* = 0.600). Best‐corrected high‐contrast monocular visual acuity (HC‐VA) and 2.5% low‐contrast visual acuity (LC‐VA), using retroilluminated Early Treatment in Diabetes Retinopathy Study (ETDRS) at 4 m distance and Sloan charts at 2 m distance, respectively, (Precision Vision, Woodstock, IL) as well as monocular color vision assessments applying the Hardy‐Rand‐Rittler pseudoisochromatic test (HRR) were acquired in 12 patients and 5 HCs. The study was approved by the local ethics committee of Charité–Universitätsmedizin Berlin (EA1/17515) and conducted in accordance with the applicable German laws and the current version of the Declaration of Helsinki. All participants gave written informed consent. All data is reported according to STROBE reporting guidelines.

### OCT

The same Spectralis SD‐OCT machine (Heidelberg Engineering, Heidelberg, Germany) with automatic real‐time (ART) function for image averaging was used in all participants.^17^ Qualitative OCT analysis was performed by an experienced ophthalmologist (O.Z.). Total macular volume (TMV) and all intraretinal layers (macular retinal nerve fiber layer; mRNFL), combined ganglion cell and inner plexiform layer (GCIP), inner nuclear layer (INL), outer plexiform layer (OPL), outer nuclear layer and external limiting membrane (ONL), combined external limiting membrane and ellipsoid zone (ELM/EZ), external limiting membrane (ELM), ellipsoid zone (EZ), retinal pigment epithelium (RPE), combined outer layers (OL; OPL to RPE) were calculated as a 6‐mm‐diameter cylinder around the fovea from a macular volume scan (25 × 30 degrees, 61 vertical B‐scan). Peripapillary retinal nerve fiber layer thickness (pRNFL) was measured with an activated eye tracker using a ring scan around the optic nerve head (12 degrees, 1536 A‐scans) or the most inner 3.5‐mm ring of a star‐and‐ring scan around the optic nerve head (12 degrees, 768 A‐scans). For patients, 21 ring scans and 28 macular scans were included for the quantitative OCT analysis after exclusions because of insufficient quality.^18^, ^19^ Segmentation of all layers was performed automatically and corrected, if necessary, by experienced raters (F.C.O. for SCA‐ATXN1, S.M. for HCs) using an in‐house–developed software^20^ based on a segmentation algorithm by Lang et al.^21^ for macular scan and a software provided by the OCT manufacturer (Eye Explorer 1.9.10.0 with viewing module 6.3.4.0; Heidelberg Engineering) for ring scans. OCT data are reported according to the APOSTEL recommendations.^22^ Clear generalized cut‐off values for pathological macular layer thinning are not yet established.

### Statistical Analyses

Group differences in age and sex between SCA‐ATXN1 and HCs were tested by χ^2^ and Wilcoxon rank‐sum tests, respectively. Group differences for OCT parameters and visual acuity assessments as well as correlations with clinical metrics were analyzed by generalized estimating equation models to account for within‐patient intereye associations of monocular measurements. All tests and graphical representations were performed with R software (V3.3.1; R Foundation for Statistical Computing, Vienna, Austria).^23^ Statistical significance was established at *P* < 0.05. Because of the exploratory character of our analyses, we refrained from corrections for multiple comparison.

### Data Availability

Data not provided in the article will be shared at the request of other investigators for purposes of replicating procedures and results.

## Results

**Figure 1:**
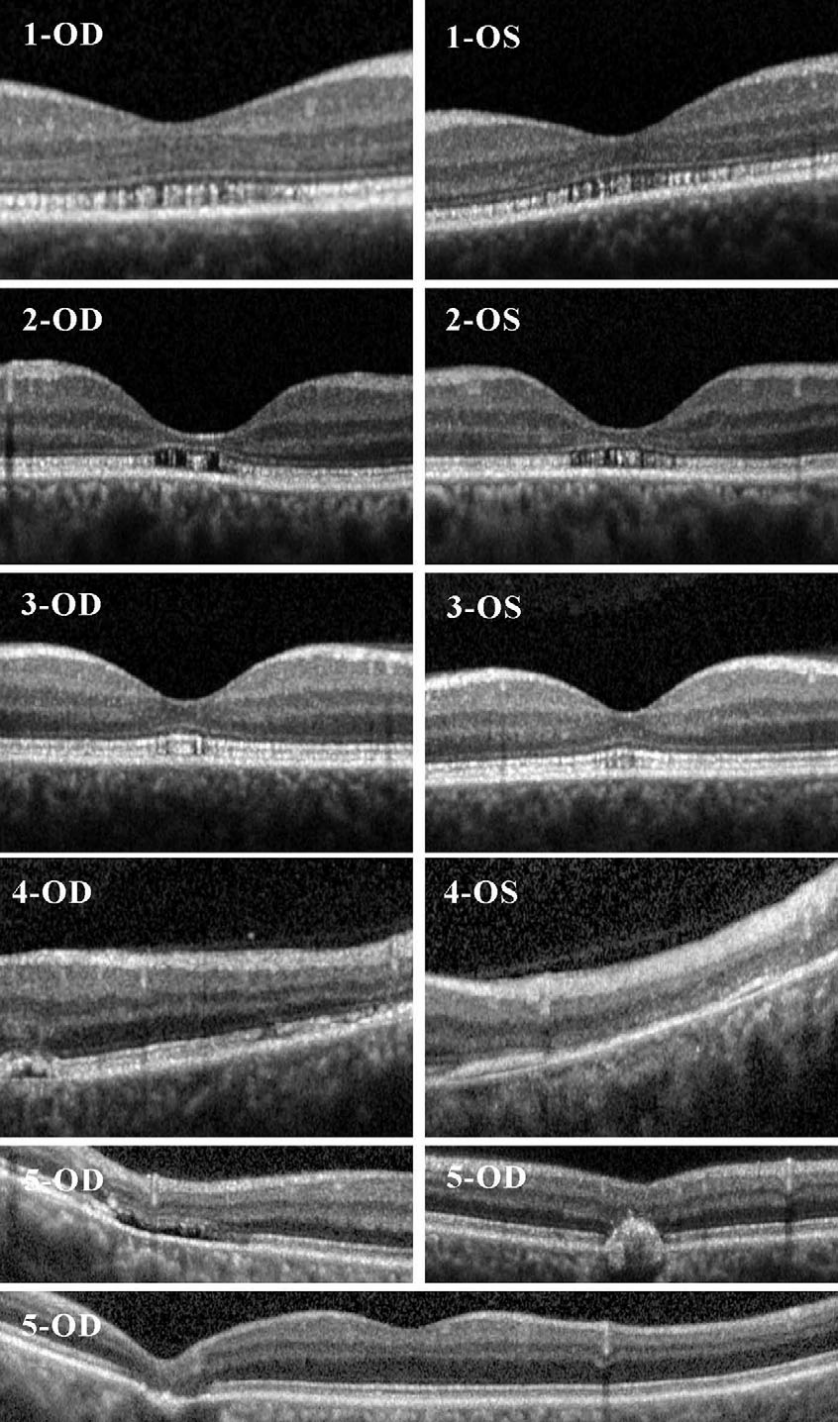
Examples of macular anomalies found in qualitative OCT analyses for SCA‐ATXN1 patients in table 3 for right eye (OD) and left eye (OS).

We found pathological disruption of EZ in 5 patients (25%; Table 2; Fig. 1). Two further patients (10%) had unspecific abnormalities (floaters, monocular epiretinal gliosis of the papillomacular bundle). The disruption of EZ was pronounced in the foveal and parafoveal area of 2 patients (Table 2; Fig. 1, Patients 1 and 2), less pronounced in the fovea of 1 patient (Table 2; Fig. 1, Patient 3), and more temporally located and less pronounced in 2 patients (Table 2; Fig. 1, Patients 4 and 5). HRR indicated an abnormal color vision in 2 patients with EZ disruption showing a mild red‐green deficiency binocularly in the first patient and monocularly in the second patient.

**Table 1:** Demographic overview for patients with SCA‐ATXN1 and age‐ and sex‐matched HCs

	HCs	SCA‐ATXN1
Subjects [N]	22	20
Age [years, mean ± SD]	47.5 ± 13.4	45.10 ± 12.37
Sex [female, N (%)]	11 (50)	9 (45)
SARA score [median (IQR)]; n = 15	—	15 (11, 17)
Time since onset [years, median (IQR)]; n = 20	—	8.5 (4.3, 11.3)
No. of CAG repeats (expanded allele) [median (IQR)]; n = 16	—	49.5 (46, 53)

IQR, interquartile range; N, number; SD, *s*tandard deviation.

**Table 2:** Case description for SCA‐ATXN1 patients with EZ disruption

	Demographics	Description of Macular Abnormality	Eye	OCT Metrics	Visual Function
1	Age 52 y Disease duration 5 to 10 y SARA score 16.0	Foveal EZ disruption	OD	GCIP 1.93 mm^3^ ELM 0.47 mm^3^ EZ 0.78 mm^3^	HC‐VA 0.00 LC‐VA 0.495 HRR Abnormal
			OS	GCIP 1.96 mm^3^ ELM 0.47 mm^3^ EZ 0.74 mm^3^	HC‐VA 0.00 LC‐VA 0.602 HRR Abnormal
2	Age 51 y Disease duration >10 y SARA score 6.0	Foveal EZ disruption	OD	Excluded from quantitative analyses	HC‐VA 0.201 LC‐VA 0.796 HRR Abnormal
			OS		HC‐VA 0.00 LC‐VA 0.495 HRR normal
3	Age 45 y Disease duration >10 y SARA score 14.5	Incipient foveal EZ disruption	OD	GCIP 1.78 mm^3^ ELM 0.50 mm^3^ EZ 0.82 mm^3^	HC‐VA –0.097 LC‐VA 0.301 HRR Normal
			OS	GCIP 1.76 mm^3^ ELM 0.48 mm^3^ EZ 0.82 mm^3^	HC‐VA –0.097 LC‐VA 0.398 HRR Normal
4	Age 54 y Disease duration 5 to 10 y SARA score 15.0	Temporal EZ disruption and drusen	OD	GCIP 1.70 mm^3^ ELM 0.45 mm^3^ EZ 0.88 mm^3^	HC‐VA 0.00 LC‐VA 0.699 HRR NA
			OS	Excluded from quantitative analyses	HC‐VA 0.201 LC‐VA 0.921 HRR NA
5	Age 35 y Disease duration < 5 y SARA score 9.0	Temporal EZ disruption, interrupted RPE contour, RPE defect	OD	Excluded from quantitative analyses	NA

N, number; NA, not available; OD, right eye; OS, left eye.

**Figure 2:**
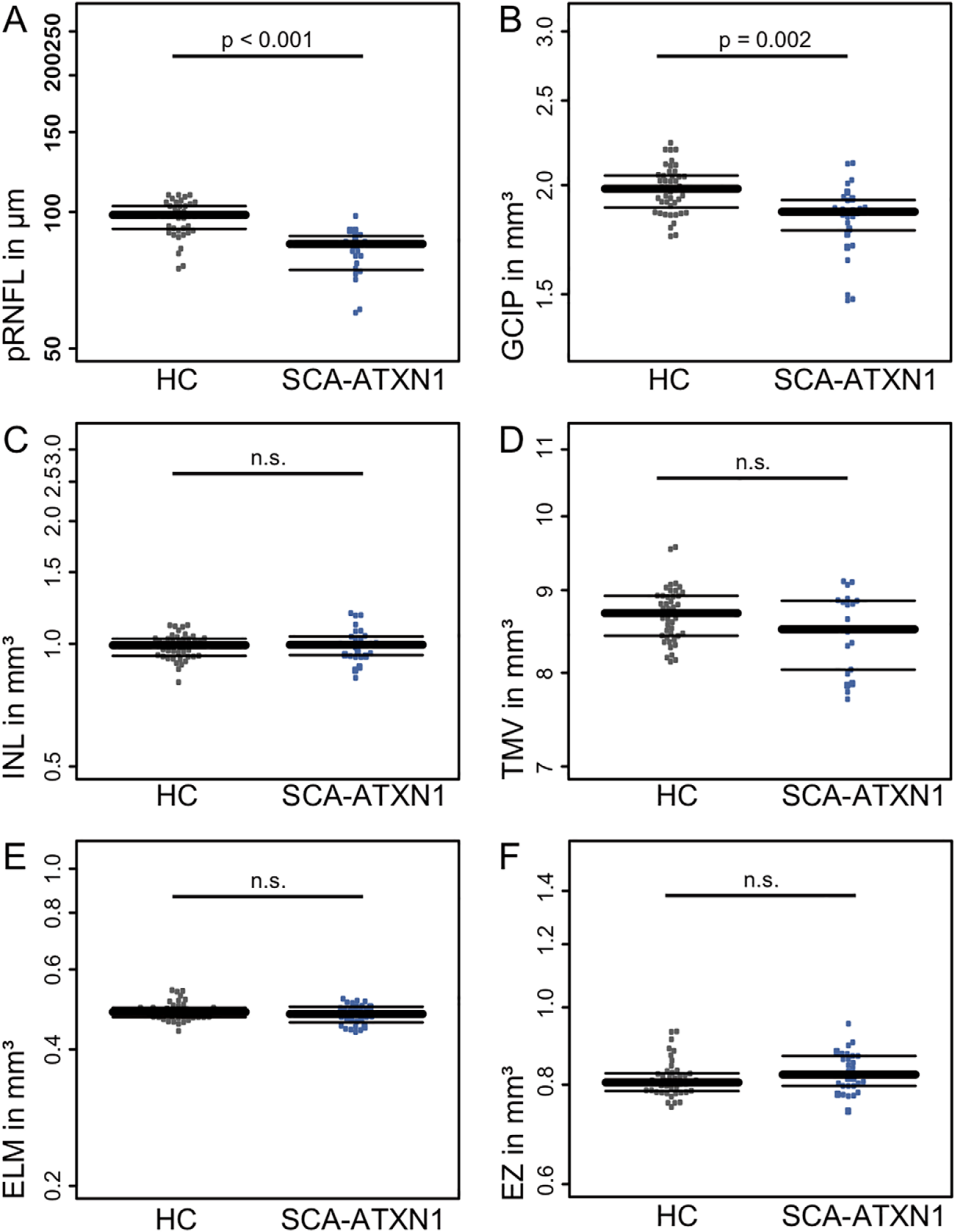
Box plots for selected OCT results in SCA‐ATXN1 (blue) and HC (gray). Patients with EZ disruption displayed as larger dots. n.s., not significant.

We then analyzed group differences in neuroretinal content between eyes from patients with SCA‐ATXN1, including those with macular anomalies, and HCs (Table 3; Fig. 2). pRNFL as the marker of axonal content in SCA‐ATXN1 was reduced compared with HCs. Compared with the device‐specific control database, 57% (N = 12) of eyes had pRNFL values within normal limits; for 19% (N = 4) of eyes, pRNFL values were borderline below, and 24% (N = 5) had pRNFL values below normal limits. Also, GCIP as a marker of ganglion cell volume was lower in SCA‐ATXN1 compared with HCs. In contrast, INL and outer retinal layers were similar between SCA‐ATXN1 and HCs (Table 3; Fig. 2). The 5 patients with disruption of EZ did visually not show signs of more pronounced retinal neuroaxonal loss (Table 2). We restrained from further statistical analysis because of the small and highly unbalanced subsets. HC‐VA as well as LC‐VA were reduced in patients with SCA‐ATXN1 compared with HCs (Table 3; Fig. 2). Because of the low sample size, we refrained from performing a statistical subgroup analysis and structure‐function correlations.

**Table 3:** Intraretinal layer quantification by OCT and visual acuity assessment in patients with SCA‐ATXN1 and HCs

	HCs [Mean ± SD]	SCA‐ATXN1 [Mean ± SD]	HC Versus SCA‐ATXN1		
			B	SE	*P* Value
OCT data					
pRNFL [μm]	97.02 ± 8.34	80.86 ± 9.49	–16.17	3.13	2.4•10^–7^
TMV [mm^3^]	8.70 ± 0.33	8.50 ± 0.46	–0.199	0.144	0.170
mRNFL [mm^3^]	1.09 ± 0.08	1.07 ± 0.14	–0.021	0.039	0.600
GCIP [mm^3^]	1.98 ± 0.12	1.84 ± 0.16	–0.144	0.047	0.002
INL [mm^3^]	0.99 ± 0.07	1.00 ± 0.09	0.015	0.025	0.560
OL [mm^3^]	4.64 ± 0.20	4.55 ± 0.36	–0.097	0.111	0.380
OPL [mm^3^]	0.70 ± 0.05	0.70 ± 0.04	0.005	0.014	0.710
ONL [mm^3^]	1.69 ± 0.15	1.63 ± 0.24	–0.070	0.067	0.330
ELM/EZ [mm^3^]	1.30 ± 0.05	1.31 ± 0.06	0.008	0.017	0.620
ELM [mm^3^]	0.49 ± 0.02	0.48 ± 0.02	–0.008	0.007	0.250
EZ [mm^3^]	0.81 ± 0.04	0.83 ± 0.05	0.017	0.014	0.220
RPE [mm^3^]	0.96 ± 0.04	0.83 ± 0.05	–0.032	0.022	0.150
Visual acuity data					
HC‐VA [logMAR]; n = 12	–0.12 ± 0.08	0.01 ± 0.10	0.134	0.040	0.001
LC‐VA [logMAR]; n = 12	0.25 ± 0.05	0.44 ± 0.16	0.185	0.046	6.0•10^–5^

IQR, interquartile range; N, number; OL, outer layers (OPL, ONL, ELM, EZ, and RPE); SD, standard deviation.

In SCA‐ATXN1 patients, time since onset showed a positive correlation with GCIP (B = 0.012; standard error [SE] = 0.005; *P* = 0.014), but not pRNFL (B = 0.480; SE = 0.424; *P* = 0.260). Furthermore, disease severity measured by SARA score was correlated neither with pRNFL (B = 0.099; SE = 0.483; *P* = 0.840) nor with GCIP (B = –0.001; SE = 0.006; *P* = 0.830). Number of CAG repeats (expanded allele) were not correlated with pRNFL nor GCIP.

## Discussion

In our study, 25% of patients with SCA‐ATXN1 displayed a distinct macular pathology, a disruption of the EZ, suggesting a retinal involvement in this disease. Furthermore, SCA‐ATXN1 patients presented with findings (pRNFL and macular GCIP loss) consistent with optic atrophy, which are pointing toward a neurodegenerative process in the optic nerve. Together with the reported visual function impairment, these structural changes should be considered as further nonataxia signs in SCA‐ATXN1.

The macular pathologies reported here are in line with three previously published cases.^24^, ^25^, ^26^ Saito et al. described an SCA‐ATXN1 patient with RPE reduction in fundus examination, subsequently reduced HC‐VA, and loss of color vision and classified the changes as pigmentary macular dystrophy similar to the alterations observed in SCA7.^25^ Vaclavik et al. described hypopigmented macular RPE and a loss of photoreceptors with a subsequent central depression of electrical potentials in 2 patients using OCT and multifocal electroretinogram.^26^ Lebranchu et al. described altered foveal lamination and abnormal spacing between RPE and external limiting membrane in 4 patients.^24^ Despite different terminology and discrepancies in examination methods, provided images in combination with descriptions highly suggest that the case reports and our study all report on the same macular pathology.

It is possible that the macular pathology is caused by a yet unrecognized pleiotropic effect of mutant *atxn1* on photoreceptors. Pathophysiological evidence for this was contributed by Fernandez‐Funez et al., who induced the expression of polyQ‐expanded ATXN1 in retinae of drosophila, which subsequently developed a progressive elimination of retinal cell bodies and axonal projections.^15^ In this drosophila study, phenotype severity was correlated with the expression level of mutant protein.^15^ In our study, EZ disruption was not associated with disease severity or pronounced retinal neurodegeneration, which may simply be a matter of sample size. Alternatively, development of maculopathy may be independent of optic atrophy in SCA‐ATXN1, which may be attributed to cell‐specific transcriptional effects of expanded ATXN1. Multiple genes associated with macular disease are located in 6p21.1, for example the genes peripherin‐2, which is critical for photoreceptor formation and maintenance, and guanyulate cyclase activator 1a, a causal gene for cone dystrophy.^24^, ^27^, ^28^ To differentiate a potential causal effect of the CAG‐repeat expansion on 6p23 from a nearby accompanying mutation, a gene analyses of the respective loci in affected patients would be necessary in the future.

Next to this first systematic description of the novel ellipsoid zone disruption maculopathy, our results solidify previous reports on optic nerve atrophy as part of the clinical picture of SCA‐ATXN1 showing pRNFL and GCIP, but not mRNFL, thinning. Of note, mRNFL is very thin and has a high intertest variability, making a significant group difference in a small sample unlikely.^20^, ^29^ Abe et al., in 1997, first reported that SCA‐ATXN1 patients showed optic nerve atrophy by fundus examination as well as color vision dysfunction and visual field abnormalities.^30^ An earlier study from our group in 2011,^14^ a study comprising several SCA genotypes by Pula et al.,^13^ and a recent case series by Nishiguchi et al.^31^ reported temporal RNFL thinning and macular neurodegeneration, respectively. The neuroaxonal damage might point toward concomitant optic neuropathy as part of a central nervous system degeneration in SCA‐ATXN1.^32^ However, data from longitudinal studies and from nonmanifest carriers are necessary to chronologically interpret retinal neurodegeneration during the disease process. To date, it remains unclear whether retinal changes are detectable in premanifest stages of disease as has been shown for some clinical and brain imaging markers.^33^

The reported changes resulted in impaired visual function in our study—with visual acuity decline as well as with color vision deficits in patients with EZ disruption. The magnitude of changes suggest that they are clinically meaningful, and at least the color vision deficits could probably be detected in clinical visual assessments using standard procedures such as Ishihara charts.^34^ Whereas the overall limited number of cases with macular pathologies preclude further analyses regarding the origin of dysfunction, the reduced visual acuity could suggest a relevance of optic atrophy for visual acuity loss. Conversely, 2 patients were suggestive of color vision deficiencies, and these 2 patients also presented with macular phenotype. This is further supported by the aforementioned case reports, also reporting, in part, color vision deficits.^24^, ^25^, ^30^

Given the rarity of SCA‐ATXN1, an important strength of the study is the comparably large sample size. However, the statistical power might have still been insufficient to confirm numerical differences in outer layers where low measurements in eyes with EZ disruption suggest a potential degenerative relevance. Further important limitations of our study include the incomplete clinical and functional (especially visual function) data and the lack of blue autofluorescence and longitudinal data to conclusively confirm a progressive retinal neurodegeneration in SCA‐ATXN1. Especially, investigations in nonmanifest carriers would be of high interest in this respect.

Our findings extend the previously established spectrum of nonataxia signs in SCA‐ATXN1 with potential relevance for diagnosis and monitoring. Within the scope of the proposed diagnostic algorithm by Rossi et al.,^35^ the ellipsoid zone disruption maculopathy may be indicative of an SCA‐ATXN1 diagnosis. However, additional research is needed to consolidate this notion, especially at disease onset when differential diagnosis is most relevant, and in comparison to other ataxias, both of which were not within the scope of our current study. With 25% of patients presenting with distinct macular changes—a disruption of the EZ—this macroscopic phenotype appears frequently enough to be considered during clinical workup. The reported changes were associated with visual dysfunction, and thus exploration and examination of the visual system should be part of patient counseling in SCA‐ATXN1. If confirmed in a longitudinal study, neurodegeneration of the afferent visual system measured by OCT may be used as a sensitive and easily accessible marker of global neurodegeneration in SCA‐ATXN1 and—in the light of approaching therapeutic candidates—even serve as outcome candidate for clinical trials.^4^, ^36^

## Author Roles

(1) Research Project: A. Conception, B. Organization, C. Execution; (2) Statistical Analysis: A. Design, B. Execution, C. Review and Critique; (3) Manuscript: A. Writing of the First Draft, B. Review and Critique.

F.C.O.: 1B, 1C, 2A, 2B, 3A

H.G.Z.: 1B, 1C, 2C, 3B

M.R.: 1C, 3B

C.B.: 1C, 3B

S.M.: 1B, 1C, 2C, 3B

J.K.: 1C, 3B

A.S.G.: 1C, 3B

A.B.: 2C, 3B

T.S.‐H.: 1C, 2C, 3B

F.P.: 1C, 2C, 3B

O.Z.: 1C, 2C, 3B

A.U.B.: 1A, 1B, 2C, 3C

## Disclosures

### Ethical Compliance Statement

The study was approved by the local ethics committee of Charité–Universitätsmedizin Berlin (EA1/17515). All participants gave written informed consent. We confirm that we have read the Journal's position on issues involved in ethical publication and affirm that this work is consistent with those guidelines.

### Funding Sources and Conflicts of Interest

F.C.O. was employed by Nocturne, unrelated to this manuscript. HGZ reports research grants from Novartis unrelated to this study. J.K. received conference registration fees from Biogen and financial research support from Krankheitsbezogenes Kompetenznetzwerk Multiple Sklerose (KKNMS), not related to this work. An.B. has received CCT devices for research from Konan Medical. F.P. reports research grants and speaker honoraria from Bayer, Teva, Genzyme, Merck, Novartis, and MedImmune and is a member of the steering committee of the OCTIMS study (Novartis), all unrelated to this work. T.S.H. received travel grants from Celgene and speaker honoraria from Roelke pharma and Biogen unrelated to this work. A.U.B. is founder and holds shares of Motognosis and Nocturne. He is named as inventor on several patent applications describing serum biomarkers for MS, perceptive visual computing for tracking of motor dysfunction, and OCT image analysis.

### Financial Disclosures for previous 12 months

T.S.H. received a travel grant from Celgene and speaker honoraria from Biogen and Bayer within the last 12 months.
